# Mutations in DNA polymerase δ subunit 1 co-segregate with CMD2-type resistance to Cassava Mosaic Geminiviruses

**DOI:** 10.1038/s41467-022-31414-0

**Published:** 2022-07-07

**Authors:** Yi-Wen Lim, Ben N. Mansfeld, Pascal Schläpfer, Kerrigan B. Gilbert, Narayanan N. Narayanan, Weihong Qi, Qi Wang, Zhenhui Zhong, Adam Boyher, Jackson Gehan, Getu Beyene, Zuh-Jyh Daniel Lin, Williams Esuma, Suhua Feng, Christelle Chanez, Nadine Eggenberger, Gerald Adiga, Titus Alicai, Steven E. Jacobsen, Nigel J. Taylor, Wilhelm Gruissem, Rebecca S. Bart

**Affiliations:** 1 Institute of Molecular Plant Biology, Department of Biology, ETH Zürich, Universitätsstrasse 2, 8092 Zürich, Switzerland; 2 Donald Danforth Plant Science Center, 975 North Warson Road, St. Louis, MO 63132 USA; 3 Functional Genomics Center Zurich, ETH Zurich and University of Zurich, Winterthurerstrasse 190, 8057 Zurich, Switzerland; 4 Department of Molecular, Cell and Developmental Biology, University of California Los Angeles, Los Angeles, CA USA; 5 Root Crops Program, National Crops Resources Research Institute, P. O. Box 7084, Kampala, Uganda; 6 Howard Hughes Medical Institute University of California Los Angeles, Los Angeles, CA USA; 7 Biotechnology Center, National Chung Hsing University, 145 Xingda Road, Taichung City, 40227 Taiwan

**Keywords:** Plant immunity, Plant breeding, Plant biotechnology

## Abstract

Cassava mosaic disease (CMD) suppresses cassava yields across the tropics. The dominant *CMD2* locus confers resistance to cassava mosaic geminiviruses. It has been reported that CMD2-type landraces lose resistance after regeneration through de novo morphogenesis. As full genome bisulfite sequencing failed to uncover an epigenetic mechanism for this loss of resistance, whole genome sequencing and genetic variant analysis was performed and the CMD2 locus was fine-mapped to a 190 kilobase interval. Collectively, these data indicate that CMD2-type resistance is caused by a nonsynonymous, single nucleotide polymorphism in *DNA polymerase δ subunit 1* (*MePOLD1*) located within this region. Virus-induced gene silencing of *MePOLD1* in a CMD-susceptible cassava variety produced a recovery phenotype typical of CMD2-type resistance. Analysis of other CMD2-type cassava varieties identified additional candidate resistance alleles within *MePOLD1*. Genetic variation of *MePOLD1*, therefore, could represent an important genetic resource for resistance breeding and/or genome editing, and elucidating mechanisms of resistance to geminiviruses.

## Abstract (web summary)

Cassava mosaic disease is caused by geminiviruses and suppresses cassava yields throughout the tropics. Here, the authors show that mutations in *MePOLD1*, encoding DNA polymerase δ subunit 1, co-segregate with *CMD2*, the major source of genetic resistance for this disease.

## Introduction

Cassava (*Manihot esculenta* Crantz) is a highly heterozygous staple root crop that feeds nearly a billion people worldwide^1^. Cassava yields are suppressed by infections with cassava mosaic geminiviruses (CMG, Family *Geminiviridae*: Genus *Begomovirus*) which collectively cause cassava mosaic disease (CMD). Eleven species of CMG are known to infect cassava across sub-Saharan Africa, the Indian subcontinent, and recently also in several countries of South-East Asia^2^. CMGs possess two circular single-stranded DNA genomes that are transmitted by the whitefly *Bemisia tabaci* and spread by farmers who plant infected stem cuttings to establish the next cropping cycle^3,4^.

**Fig. 1:**
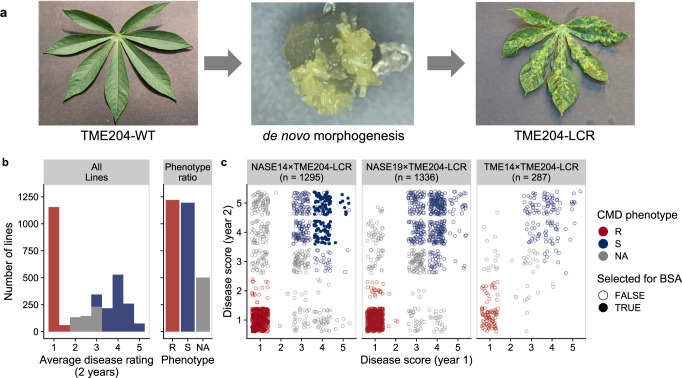
CMD2 type cassava varieties lose resistance upon de novo morphogenesis. **a** Left—TME204-WT CMD2-type plants challenged with cassava mosaic geminivirus remains symptom free. Middle – embryogenic structures arise from tissue culture-induced de novo morphogenesis. Right—Regenerated plant shows classic mosaic symptoms after virus challenge. **b** F1 populations derived from heterozygous resistant parents (NASE14, NASE19, TME14) crossed with susceptible loss-of-CMD2-resistance (LCR) line. Plants were grown and phenotyped in the field in Uganda and scored for disease over two years on a 1-5 disease score. The disease rating distribution across all populations segregates at 1:1 (χ^2^ *p*-value = 0.5263; *R* = 1291, *S* = 1259). **c** In the NASE14xTME204-LCR population, 125 resistant and 125 susceptible lines with consistent phenotypes over the 2 years were selected for bulk segregant analysis (BSA) mapping (solid circles).

Understanding genetic sources for resistance to geminiviruses is critical to securing yields for cassava farmers. Three types of resistance to CMGs have been described in cassava as CMD1, CMD2, and CMD3^5,6^. In all cases, the genes responsible for resistance and their modes of action remain unknown. CMD2-associated resistance, which was discovered in landraces collected across West Africa, is a dominant, single genetic locus located on Chromosome 12^7–10^. We reported previously that CMD2-type resistance is lost when plants are regenerated through de novo morphogenesis in tissue culture^11^ (Fig. 1a). While the loss of CMD2 resistance (LCR) occurs consistently in this manner in multiple landraces, LCR was not observed in varieties developed through breeding programs^12^.

Here, we demonstrate that the CMD2 and LCR phenotypes have a genomic basis and co-localise on the cassava genome. Using whole-genome sequencing and genetic variant analysis (WGS-GVA), we identified independently evolved, nonsynonymous single nucleotide polymorphism (SNP) variants in *DNA polymerase delta subunit 1* (*MePOLD1*) that segregate with CMD resistance. In parallel, we developed and phenotyped large populations to fine-map the CMD2 locus to 190 kb and found that SNPs within *MePOLD1* are the only observed genetic or epigenetic change within this region. Virus-induced gene silencing of *MePOLD1* in a susceptible cassava variety led to a recovery phenotype typically observed in resistant varieties. By screening the cassava germplasm data, we identified additional alleles in *MePOLD1* that correlate with resistance phenotypes. Moreover, we show that a premature stop codon in a *MePOLD1* allele that co-segregates with resistance results in susceptibility to CMD. Our study indicates that the mutations in *MePOLD1* likely mediate CMD2-type resistance, with further work necessary to understand the underlying mechanism.

## Results and discussion

### *Loss of CMD2 resistance* (LCR) and CMD2 co-segregate and *CMD2*-mediated resistance may be a chimeric trait

Epigenetic somaclonal variation is well known to produce phenotypic changes in plants regenerated from in vitro cultures^13,14^. We hypothesised, therefore, that the LCR phenotype is caused by culture-induced epigenetic changes at the CMD2 locus. Single-cytosine resolution epigenome-wide association studies (EWAS) were performed on multiple cassava plant lines before, and after, in vitro morphogenesis. While methylation changes were found across the genome, no consistent methylation changes were observed within the CMD2 locus (Supplementary Fig. 1, Supplementary Table 1).

We therefore investigated the relationship between the CMD2 and LCR phenotypes by generating three large mapping populations derived from tissue culture regenerated, CMD susceptible plants (TME204-LCR) crossed with resistant varieties heterozygous for CMD2 (NASE14, NASE19, TME14^8,15^). Field phenotyping was performed over two years at a high CMD pressure location in Uganda, and progeny lines assessed for resistance or susceptibility to CMD (Fig. 1b, Supplementary Data 1). Resistance segregated at 1:1 ratio (Fig. 1b, across all populations, χ^2^ *p*-value = 0.5263, *R* = 1291, *S* = 1259), indicating that the dominant wild-type allele of CMD2 is sufficient to restore resistance, and that the CMD2 and LCR phenotypes are caused by a single locus. If LCR results from a somaclonal epiallele, then passage of CMD-resistant F_1_ progeny through morphogenesis would result in the LCR phenotype. However, three independent, resistant F_1_ progeny retained resistance through three consecutive cycles of somatic embryogenesis and plant regeneration, indicating that sexual propagation stabilises CMD2-type resistance and prevents LCR from occurring after de novo morphogenesis in tissue culture (Supplementary Fig. 2). These results indicate that the CMD2 and LCR traits have a genomic basis. We postulate that spontaneous mutation(s) causing CMD2 resistance occurred in the meristems of field-grown West African landraces and became fixed as periclinal chimeras (Supplementary Fig. 3). The subset of mutated cells continued to develop into asymptomatic branches, which would be selected and maintained by farmers through clonal stem propagation. Development and propagation of beneficial periclinal chimeras is known and common in other crop species^16–18^. Loss of resistance to CMD would be explained if de novo morphogenesis occurs from cell layers that do not carry the resistance allele. Gametes are typically derived from cells within the L2 layer of the meristem^19^, thus if L2 cells carried the dominant *CMD2* mutation it would be transmitted to the next generation in a Mendelian manner. The resulting progeny plants would not be chimeric for the resistance allele and, as we report here, would not lose resistance to CMD after in vitro morphogenesis (Supplementary Fig. 3).

### A nonsynonymous SNP in *MePOLD1* co-segregates with CMD2 resistance within a 190 kb fine-mapped locus

**Fig. 2:**
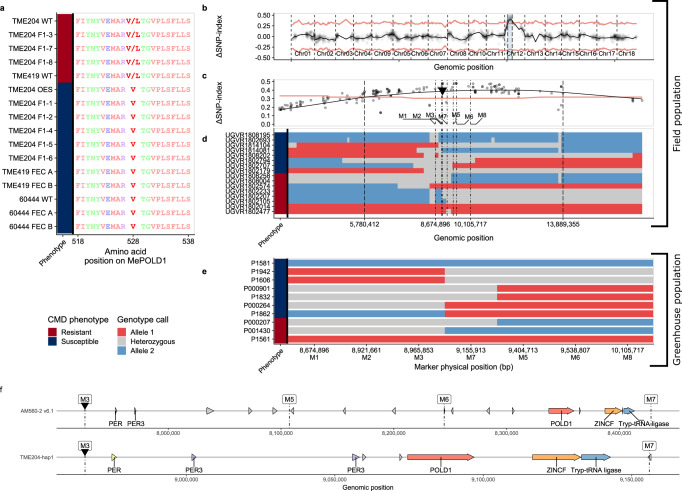
Whole-genome sequencing and genome variant analysis (WGS-GVA) and fine mapping reveal nonsynonymous SNPs in MePOLD1 that segregate with resistance. **a** TME204-WT and F1 progeny, TME419-WT, 60444-WT and TME204, TME419 and 60444 plants regenerated from tissue culture (FEC) were tested for resistance and susceptibility. TME204 WT, F1-3, F1-7, F1-8, and TME419-WT plants had CMD2 resistance while all other plants were susceptible to ACMV infections. The resistance phenotype is indicated on the left bar (Red – Resistant; Blue – Susceptible). A haplotype 1-restricted G to C transversion in the TME204 MePOLD1 gene at location 9,081,215 bp causes a heterozygous V528L mutation in MePOLD1. Two large (*n* ≈ 1000) F_1_ mapping populations derived from NASE14×TME204-LCR were used to fine-map CMD2 (**b**–**e**). **b** An in silico bulk segregant approach was performed using the field phenotyping and genotyping by sequencing (GBS) data (Fig. 1c). The tricube-smoothed allele frequency enrichment (ΔSNP-index) across the TME204-hap1 assembly. In (**c**) and (**d**) the red line denotes the 95% confidence interval. The highlighted region on Chr12 defines the significantly linked CMD2 region. **c** Enlargement of the CMD2 locus mapping results. Each point represents a SNP and its corresponding ΔSNP-index. The dashed lines indicate the borders of the mapped locus between ~5–13 Mb. The previously reported associated marker from Rabbi et al. is indicated by black arrow^9^. **d** Examining the GBS SNP data from individual recombinants within the locus improves the mapping resolution to ~300 kb. Genotypes are extended downstream until the next SNP called. Two non-recombinant homozygous resistant and susceptible lines are added as a control (top and bottom). Based on the location of the mapped locus, and the previously identified GWAS marker, KASP markers (M1-8) were developed for fine mapping (positions denoted by dot-dash lines in (**c**) and (**d**). **e** A second fine-mapping population was phenotyped in the greenhouse using a virus-induced gene silencing-based infection assay. Recombinants within the region place CMD2 in the 190Kb interval between markers M3 and M7. Lines P1581 and P1561 are non-recombinant susceptible and resistant controls, respectively. In (**c**) and (**e**) the genotype at each SNP or marker is indicated by the colour (Allele 1, Red, linked to Resistance; Allele 2, Blue, linked to Susceptibility). The resistance phenotype is indicated on the left bar as above. **f** Genomic rearrangements within the fine-mapped CMD2 locus introduce new gene candidates.

We combined WGS-GVA with fine-mapping to identify *CMD2* and further understand the LCR trait. WGS-GVA has been used to understand the genetics behind rare human diseases, where causal variants shared by multiple individuals or families are revealed by comparison of WGS from sick and healthy individuals^20,21^. We performed WGS-GVA to identify genetic changes in three CMD-resistant and five susceptible F_1_ plants (Supplementary Data 2). A filtering approach (Methods, SNP analysis) identified 405 SNPs segregating with the resistance phenotype in these individuals (Supplementary Data 3). We hypothesised that if the LCR phenotype is indeed caused by the absence of a resistance-inducing mutation within *CMD2*, then wild-type resistant TME204 should share variants with resistant F_1_ individuals, while susceptible LCR lines would not. Of the 405 SNPs identified in the resistant F_1_ progeny, only one nonsynonymous SNP is heterozygous in the genome of resistant TME204 and absent in the genome of susceptible TME204-LCR plants. This observation is consistent with the hypothesis that CMD resistance is a chimeric trait in landraces and that passage through culture-induced embryogenesis leads to loss of chimerism and CMD2 resistance. The SNP is located in the coding sequence of *MePOLD1* (Manes.12G077400) and changes valine to leucine (V528L) (Fig. 2a). EWAS confirmed that *MePOLD1* has no DNA methylation differences in resistant and susceptible genotypes (Supplementary Fig. 1d).

We also pursued fine-mapping to pinpoint the CMD2/LCR genomic location. The recently released haplotype resolved genome assemblies of CMD2-resistant African cultivars TME7^22^ and TME204^23^ were leveraged to perform in silico bulk segregant analysis (BSA) (based on Takagi et al.^24^ and Mansfeld and Grumet^25^) to map CMD2 resistance. First, F_1_ progeny were screened in the field in Uganda and genotyped with GBS (Fig. 1b, Supplementary Data 1). These data co-localise the CMD2/LCR locus with the previously identified CMD2 locus^9^, placing it on Chromosome12 between 5 and 13 Mb of the TME204 haplotype 1 assembly^23^ (Fig. 2b). We identified recombinants within this region using SNP calls from individual samples, thus narrowing the CMD2/LCR-locus to roughly 300 kb (Fig. 2c, d). To more accurately fine-map the locus, kompetitive allele specific PCR (KASP) markers were developed bracketing this region (Fig. 2c–f, Supplementary Fig. 4, and Supplementary Data 4). Approximately 1,000 F_1_ individuals derived from a NASE14×TME204-LCR cross were genotyped and then phenotyped in the greenhouse (Supplementary Data 5) using a previously described virus-induced gene silencing (VIGS)-based infection assay^26^. We identified 64 (~6.57 cM) recombinants between markers M1 and M8 and further screened those individuals using three additional markers (M3, M5, M7). This allowed the identification of recombinants which narrowed the CMD2/LCR locus to 190 kb, between M3 (8,965,853 bp) and M7 (9,155,913 bp) in the TME204-hap1 assembly^23^ (Fig. 2e, f).

The marker order in both TME7 and TME204^22,23^ assemblies is different than in the AM560-2 v6.1 assembly^27^, suggesting a translocation or assembly error in the region which may have complicated previous efforts to find *CMD2* (Fig. 2f). The newly defined fine-mapped locus consists of eight annotated genes, including several peroxidase genes that were previously proposed as CMD2 candidate genes^9,10,28^ and *MePOLD1* (Fig. 2f). Differential gene expression analyses between susceptible and resistant individuals revealed no significant differences for genes found within this region (Supplementary Fig. 5). Nucleotide level comparison of WGS data revealed that the V528L SNP in *MePOLD1* was the only genetic change between these recombinant lines.

### Targeting *MePOLD1* with VIGS in a susceptible cassava variety leads to a recovery phenotype

**Fig. 3:**
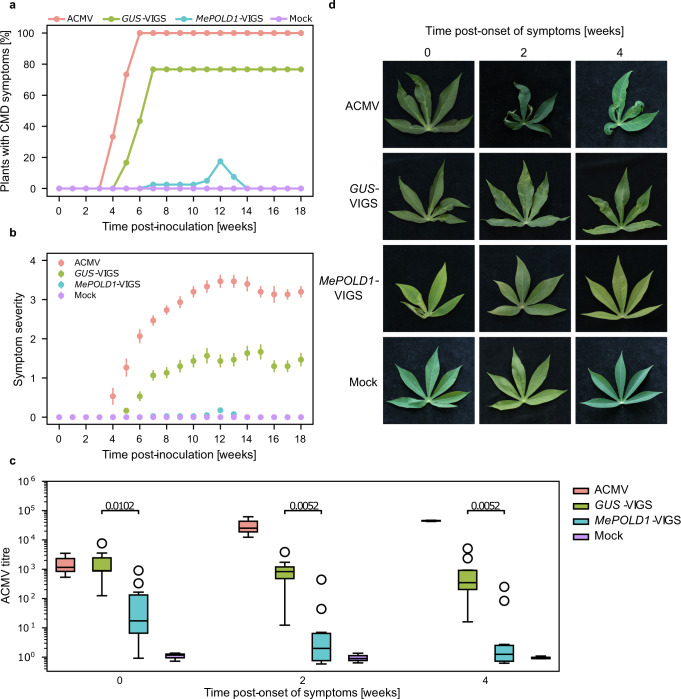
VIGS targeting of *MePOLD1*. CMD-susceptible cassava 60444 recovers from ACMV infection when *MePOLD1* is downregulated by VIGS. **a** Percentage of symptomatic 60444 plants and (**b**) CMD symptom severity according to Fauquet and Fargette, 1990^56^ 18 weeks post-inoculation: ACMV (*n* = 15), *GUS*-VIGS (*n* = 30), *MePOLD1*-VIGS (*n* = 40), and Mock (*n* = 15). Bars show standard error. **c** Quantification of ACMV titre post-onset of CMD symptoms after inoculation with ACMV (*n* = 3), *GUS*-VIGS (*n* = 10), *MePOLD1*-VIGS (*n* = 10), and Mock (*n* = 3). Week 0 is the first onset of symptoms detected on individual plants. The data were presented as standard boxplots (the box encompasses Q1–Q3, the median is shown as a central horizontal line within the box, and the whiskers cover the data within ±1.5 IQR). Significance was determined using a two-tailed, Mann–Whitney-*U* test adjusted with the Benjamini–Hochberg procedure. **d** Representative images of the leaves used for experiments shown in panels (**a**–**c**).

Taken together, these data suggest that variation within the *MePOLD1* CDS underlie CMD2-type resistance. Finding a nonsynonymous SNP by WGS-GVA in the precisely mapped CMD2 locus by chance is statistically improbable (*P* = 6.1 × 10^−4^, Monte Carlo simulation, *n* = 100,000). Components of the DNA polymerase complex have previously been reported to be required for susceptibility to geminiviruses^29–33^. To understand if this holds true for cassava, we targeted *MePOLD1* for downregulation in the CMD-susceptible cassava variety 60444 using VIGS (*MePOLD1*-VIGS)^34^. After inoculation with *MePOLD1*-VIGS, only 25% (*n* = 40) of 60444 plants showed symptoms of infection compared to plants infected with *GUS*-VIGS (76.7%, *n* = 30) and *African cassava mosaic virus* (ACMV) (100%, *n* = 15). CMD symptom severity after *MePOLD1*-VIGS was also reduced in infected plants of 60444 (Hypergeometric Test, *P* < 0.05, *n* = 40, Fig. 3a, b) and virus titre was significantly lower when compared to plants inoculated with control VIGS constructs or unmodified ACMV (Fig. 3c). Importantly, plants of 60444 that displayed CMD symptoms after inoculation with *MePOLD1*-VIGS underwent a recovery phenotype typical of CMD2 resistance and atypical for this highly CMD-susceptible variety (Fig. 3d). While the phenotypic result of *MePOLD1-*VIGS was clear, we did not observe a significant downregulation of *MePOLD1* mRNA levels in 60444 inoculated with *MePOLD1*-VIGS vectors (Supplementary Fig. 6). This may be because *MePOLD1* is already expressed at very low levels in leaf tissues (Supplementary Fig. 7^35^), or reflect inherent complexity associated with using a geminivirus-based vector to down-regulate a gene required for geminivirus replication (Supplementary Fig. 8). In a similar experiment in *Nicotiana benthamiana* that used the RNA virus Tobacco rattle virus (TRV) as the VIGS system, a significant reduction in *Tomato yellow leaf curl virus* (TYLCV) accumulation and virus-induced downregulation of *POLD* were observed^31^. Together, our results demonstrate that *MePOLD1*-VIGS is sufficient to provide CMD resistance, although further work is necessary to understand why an RNAi-mediated downregulation of *MePOLD1* expression was not observed.

### Additional nonsynonymous SNPs in *MePOLD1* correlate with CMD resistance

**Fig. 4:**
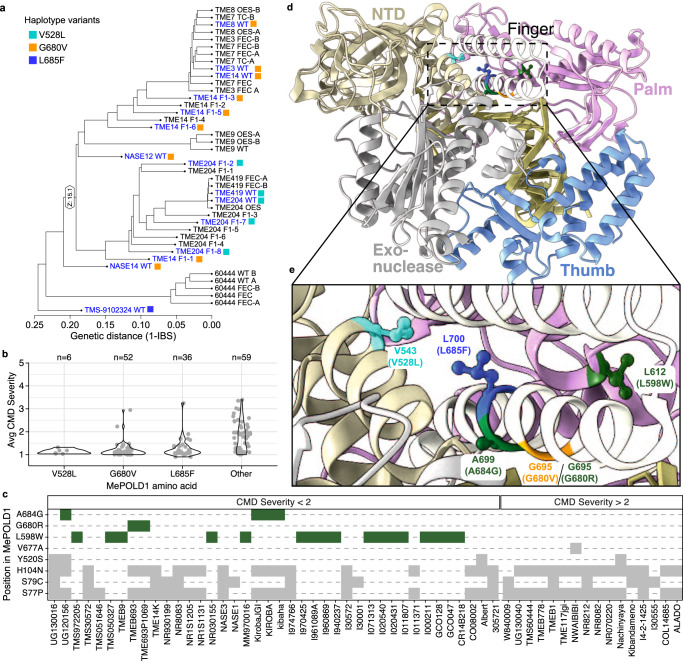
Nonsynonymous SNPs in *MePOLD1*. **a** Dendrogram of *Manihot esculenta* cultivars analysed by whole genome sequencing. Nonsynonymous SNPs (nsSNPs) in *MePOLD1* of various cultivars segregate with CMD2 resistance. Names of resistant cultivars are in blue and harbour either the V528L (cyan), G680V (orange), or L685F (blue) mutation. **b** Average CMD severity across a diverse set of cassava cultivars from the HapMapII population^37^ that have either one of the three mutations from (**a**) or an unknown nsSNP in *MePOLD1* ("Other”). **c** Identity of all nsSNPs in *MePOLD1* of varieties from the “Other” category in (**b**). Varieties are split by CMD severity score, where less than 2 and above 2 are resistant and susceptible, respectively. In green are the nsSNPs found only in cultivars with CMD severity scores below 2; all other nsSNPs are in grey. **d** Three-dimensional structure of *S. cerevisiae* POLD1 (PDB: 3IAY) with corresponding MePOLD1 mutations highlighted; V528L in cyan, G680V in orange, and L685F in blue. Additional residues identified in (**c**), L685F and L598W, are in green. Residue name and position in ScPOLD1 are noted and the corresponding information for MePOLD1 is in parentheses. POLD1 functional domains, N-terminal (beige), exonuclease (grey), and structural motifs of the polymerase domain, palm (pink), fingers (white), and thumb (blue), are highlighted. **e** Zoomed in view of the 3D structure centred on the mutated residues found in MePOLD1.

We next investigated the *MePOLD1* coding sequence of additional CMD-resistant cultivars using WGS-GVA and/or Sanger sequencing (Fig. 4, Supplementary Data 3, Supplementary Data 6). The V528L allele present in TME204 was also observed in TME419 (Fig. 2a, Fig. 4), consistent with these landraces being closely related, and both collected from farmers’ fields in Togo/Benin^36^. While other resistant varieties did not contain the V528L allele, two additional nonsynonymous SNPs were identified within *MePOLD1* (G680V in TME3, TME8, TME14, NASE12 and NASE14 and L685F in TMS-9102324) (Fig. 4, Supplementary Fig. 9). These results suggest that several distinct *MePOLD1* alleles may explain CMD2 resistance. We also queried publicly available re-sequencing data of diverse cassava germplasm^27,37^ and cross referenced these varieties for CMD severity phenotype data available at CassavaBase^38^. Of the 241 accessions with re-sequencing data, 153 have associated CMD susceptibility scores. *MePOLD1* SNPs were identified in 94 of the resistant accessions (CMD score of less than 2 out of 5). Specifically, 6, 52, and 36 accessions harbour V528L, G680V, or L685F, respectively. (Fig. 4b). Analysis of the remaining 59 varieties identified three additional nonsynonymous SNPs in *MePOLD1* unique to accessions with CMD severity scores below 2: L598W, G680R, and A684G; found in 17, 2, and 4 samples, respectively (Fig. 4c). In every case, across 117 samples in which MePOLD1 variants were identified, the putative resistance allele is observed in the heterozygous context, suggesting that these amino acid changes might be deleterious if homozygous. Indeed, an EMS mutant in Arabidopsis POLD1 (at position A684 in MePOLD1; Fig. 4c) is hypomorphic and lethal at 28 °C^39^. Five of the six mutations identified in MePOLD1 (V528L, G680V, G680R, A684G, L685F) are immediately adjacent to the R696-E539 (MePOLD1: R681-E524) salt bridge between the finger and N-terminal domains described in yeast POLD (Fig. 4d, e). Mutations disrupting this salt bridge have been shown to result in decreased polymerase activity and fidelity^40,41^. Furthermore, a homozygous R696W mutation is lethal in yeast and is associated with oncogenesis in humans^41^.

### Loss of a *MePOLD1* allele that co-segregates with CMD resistance leads to susceptibility

The above data suggest a model wherein *MePOLD1* is a susceptibility factor involved in cassava geminivirus replication and that nonsynonymous mutations within *MePOLD1* lead to CMD2-type resistance. We applied this model to an unexplained observation. The CMD-resistant NASE14 parent from the mapping populations is heterozygous for the G680V mutation. NASE14 (the line formerly known as MM96/4271) was developed in a breeding program at the International Institute for Tropical Agriculture^42^ and does not lose resistance after passage through culture-induced morphogenesis^12^. Unexpectedly, in a previous experiment where NASE14 was used to generate multiple transgenic lines, we observed a single line 5001-NASE14-#41 that had lost resistance to CMD^43^. To understand this outcome, targeted Sanger sequencing of *MePolD1* was performed on 5001-NASE14-#41^43^ that had lost CMD2 resistance. The result confirmed that this line retained the heterozygous nonsynonymous SNP that would lead to the G680V mutation in the MePOLD1 protein, characteristic of the resistant NASE14 cultivar. However, examining the cloned, full-length CDS revealed the presence of an additional heterozygous SNP not present in WT NASE14 that introduces a premature stop codon at amino acid position 574 within the resistance allele (Supplementary Fig. 10). Thus, transgenic event 5001-NASE14-#41 contains a susceptible version of *MePOLD1*, but lacks its original functional resistance allele, which would explain its acquired susceptibility to infection by CMGs. This spontaneous knock-out of the resistance allele provides further strong evidence that mutations in *MePOLD1* explain CMD2 type resistance in cassava.

Collectively, our data indicate that amino acid changes near the active centre of MePOLD1 likely mediate the dominant CMD2-type resistance. No other genetic or epigenetic changes were observed within the fine-mapped CMD2 locus that also segregate with resistance. Several dominant resistance genes for plant viruses have been reported, most of which belong to the NBS-LRR class of proteins^44^. MePOLD1 likely represents an unexpected, novel type of resistance protein in plants. Evidence suggests that these *MePOLD1* alleles have been selected as chimeric clonal variants multiple times by West African farmers. Due to its monogenic, dominant nature, CMD2 is now favoured in breeding programs in Africa, India, and South-East Asia^8^. Mutations in POLD1 predispose humans and mice to a range of cancers, especially mutations that specifically affect the proofreading activity or dNTP selectivity of the enzyme^45^. It is possible that the identified mutations in MePOLD1 may similarly introduce replication errors in geminiviruses, impairing replication efficacy and thereby reducing virus load in the host plant. This hypothesis is supported by the co-localisation of MePOLD1 mutations to those in yeast and humans known to decrease DNA replication activity, and accuracy^40,41,45^. We cannot exclude, however, that the MePOLD1 mutations weaken or block interactions with the virus replication-enhancer protein AC3, which interacts with subunits of POLD^31^. CMD2 resistance has remained robust in farmers’ fields over at least three decades. However, some caution for overreliance on CMD2 is presented here with evidence that yields and livelihoods for millions of cassava farmers are being secured by a few SNPs in one gene. The identification of mutations in *MePOLD1* as the likely cause for CMD2-type resistance will facilitate the production of CMD-resistant cassava varieties by SNP-assisted breeding or genome editing to introduce the identified SNPs into susceptible cultivars and provides the opportunity to further elucidate mechanisms of resistance to geminiviruses in cassava and other crops.

## Methods

### Plant lines, mapping populations and disease scoring

For detailed descriptions of each plant line used in this study, see Supplementary Table 1 and Supplementary Data 1 and 2. TME204-LCR was described previously^46^.

A program was conducted in Uganda during the 2017/2018 cropping season to perform controlled crosses between CMD susceptible cultivar TME204-LCR and the three CMD resistant wildtype cassava varieties TME14, NASE14, NASE19 following the standard procedures described by Kawano (1980)^47^ and Hahn et al., (1980)^48^. During the pollination period, special care was taken to cover mature flowers with pollination bags 2–3 days before and after pollination. A total of 7,200 botanical seeds were harvested from mature fruits within three months after pollination and stored in paper bags for approximately three weeks to break dormancy. All seeds were planted in field-conditioned nursery beds and 4300 resultant seedlings transplanted to a field at six weeks of age and allowed to grow under natural field conditions for 12 months. The field trials were conducted at Namulonge, central Uganda, which is a hotspot for cassava mosaic disease with high whitefly vector populations. CMD-symptomatic plants of local cultivar Bao were planted as spreader rows to augment field inoculation of CMGs. To achieve phenotyping, monthly CMD severity was scored starting one month after transplanting seedlings, and recorded on a 1–5 scale^49^ where 1 = no symptoms; 2 = mild chlorotic pattern over the entire leaf although the leaf appears green and healthy; 3 = moderate mosaic pattern throughout the leaf, narrowing and distortion in the lower one-third of leaflets; 4 = severe mosaic, distortion in two-thirds of the leaflets and general reduction in leaf size; and 5 = severe mosaic distortion in the entire leaf. The final CMD severity data recorded at the crop age of 11 months were used for subsequent analyses. The disease rating distributions of the entire ~3000 individual population were plotted to assess if epistatic segregation ratios could be observed. To ensure robust resistance phenotype descriptions, only plants with a two-year mean disease rating of less than 2 were defined as resistant and lines with consistent disease ratings above 3 in both years were denoted as susceptible. The 1:1-R:S ratio was tested using a chi-square test (chisq.test function) in R.

A similar crossing program was established at Kandara, Kenya in which TME204-LCR was crossed with the two CMD resistant wildtype cassava varieties TME14 and NASE14. Resulting seeds were collected and shipped to DDPSC, St Louis, USA.

### Epigenome-wide association studies (EWAS)

Whole-genome methylation of TME7 and TME204 samples were prepared with Bisulfite Kit (Qiagen, Germantown, Maryland, USA) and enzymatic Methyl-Seq kit (New England BioLabs, Ipswich, Massachusetts, USA), respectively. Genomic DNA from samples in the TME7 background was end-repaired and ligated with TruSeq DNA single adapters (Illumina) using a Kapa DNA HyperPrep kit (Roche). Adapter-ligated DNA was converted with an EpiTect Bisulfite Kit (Qiagen). Converted DNA was PCR-amplified by MyTaq polymerase (Bioline) for 12 cycles. EM-seq libraries for samples in TME204 background were prepared from sheared DNA using an enzymatic Methyl-Seq kit following manufacturer instructions (New England BioLabs) with 6 PCR cycles^50^. The libraries were run on D1000 ScreenTape (Agilent) to determine quality and size, and then purified by AMPure XP beads (Beckman Coulter). Library concentrations were measured with a Qubit dsDNA Broad-Range Assay kit (ThermoFisher). Libraries were sequenced on a HiSeq 2500 or NovaSeq 6000 sequencer (Illumina).

WGBS and EM-seq reads were mapped to haplotype 1 and haplotype 2 genomes of TME204 by BSMAP (v2.90) allowing 0 mismatches and one best hit (-v 0 -w 1)^51^. Duplicated reads were removed with SAMtools (v1.3.1)^52^. Reads with three or more consecutive methylated CHH sites were considered as unconverted reads and removed in the following analysis. The conversion rate was estimated by calculating methylation level of the chloroplast genome. DNA methylation level at each cytosine was calculated by number of methylated C vs. total C and T count. Differentially Methylated Cytosines (DMCs) were identified by methdiff.py in BSMAP^51^ where differences in CG, CHG, and CHH methylation were at least 0.3, 0.2, and 0.1, respectively. Methylation levels of DMCs of each sample versus three TME7 and one TME204 wildtype were merged as a consensus DMCs table. Methylation levels of each sample in DMCs table were subjected to one-way ANOVA test by comparing seven resistant vs. seven susceptible samples to calculate *p*-value of each DMC. Manhattan plot of *p*-value were generated by R package qqman^53^. Methylation track files were visualised with Integrative Genomics Viewer (IGV, v3.0)^54^.

### CMD resistance across cycles of somatic embryogenesis

The three CMD-resistant F1 progeny lines, NASE14×TME204-LCR.82, NASE14×TME204-LCR.73, and NASE14×TME204-LCR.16 were established, and micropropagated in tissue culture. Organised somatic embryos (OES) were induced from leaf explants and plants regenerated to produce Cycle 1 OES-derived plants^55^. This process was repeated with Cycle 1 OES-derived plants to produce Cycle 2 OES-derived plants, and again to generate Cycle 3 OES-derived plants for each of the three F1 progeny lines. Regenerated plants were established in the greenhouse^55^ and inoculated with *East African cassava mosaic virus* (EACMV-KE2) isolate K201 as described previously^26^. Ten plants were inoculated from each cycle of OES-derived plants for all three progeny and assessed for the development of CMD leaf symptoms over a period of 90 days using a 0–5 visual scoring method^56^. At 51 days after inoculation plants were ratooned (cut back) and a new round of CMD symptoms scored on leaves produced by shoot regrowth to confirm the original phenotype.

### Whole genome sequencing and genomic variant analysis (WGS-GVA)

Illumina sequencing: Leaf material was collected from 42 cassava genotypes and friable embryogenic callus (FEC) material from two cassava genotypes (Supplementary Table 3) for whole-genome Illumina sequencing. DNA was extracted using the DNeasy Plant Mini Kit (QIAGEN, Germany). DNA samples were sent to the Functional Genomics Center Zurich (FGCZ) for Illumina sequencing. DNA libraries were prepared using the Illumina TruSeq Nano DNA High Throughput Library Prep Kit (20015965), following the manufacturer’s protocol (Illumina, San Diego, California). Libraries were sequenced using an Illumina NovaSeq system for 2 × 151 cycles, according to the manufacturer’s instructions (Illumina, San Diego, California). On average 100X Illumina paired-end (PE) data were collected per sample.

Pre-processing and mapping of reads: Quality control and Bowtie2 alignment of the Illumina PE reads were performed using data analysis workflows in the R-meta package ezRun (https://github.com/uzh/ezRun), managed by the data analysis framework SUSHI4, which was developed and maintained by FGCZ. Technical quality was evaluated using FastQC version 0.11.7. Possible contaminations were screened using FastqScreen version 0.11.1 against a customised database in SUSHI, which consists of SILVA rRNA sequences (https://www.arb-silva.de/), UniVec (https://www.ncbi.nlm.nih.gov/tools/vecscreen/univec/), refseq mRNA sequences and selected genome sequences (human, mouse, Arabidopsis, bacteria, virus, phix, lambda, and mycoplasma) (https://www.ncbi.nlm.nih.gov/refseq/). Illumina PE reads were pre-processed using fastp (v0.20.0), where sequencing adapters and low-quality ends (<Q20) were trimmed. Trimmed reads passing the filtering criteria (average quality > = Q20, minimum length > =18 bp) were aligned to the *Manihot esculenta* TME204 genome (V1.0, FGCZ) using Bowtie2 version 2.3.2 with the --very-sensitive option. PCR-duplicates were marked using Picard version 2.9.0. Read alignments were comprehensively evaluated using the mapping QC app in SUSHI, in terms of different aspects of DNA-seq experiments, such as sequence and mapping quality, sequencing depth, coverage uniformity and read distribution over the genome.

Freebayes Variant Calling: Multi-sample, frequency-based calls for all variants with allele frequency above 20% were generated using the freebayes-parallel script in freebayes (v1.2.0-4- gd15209e), with 24 threads of freebayes running in parallel across regions of 100 kb in the reference genome. Dendrogram and underlying relatedness analysis of SNPs using identity-by-descent (IBD) measures was performed using the R/Bioconductor Package SNPRelate (v 3.13).

SNP analysis: To find potential SNPs, a custom python script (https://github.com/pascalschlaepferprivate/filter_vcf) parses the VCF file produced by freebayes, computes total coverage of the SNP, and then absolute and relative read coverage of all SNP variants. Four groups of genotypes can be defined to filter SNP results in the VCF file: ingroup (genotypes that show a SNP variant of interest), outgroup (genotypes that do not show SNP variant of interest), facultative ingroup (genotypes that may show SNP variant of interest), and facultative outgroup (genotypes that may not show SNP variant of interest). Seven parameters are given to the script. Minimal total read coverage (mtrc) defines the minimum number of reads (all variants included) that each genotype has to show to be qualified for further filtering. Minimum relative read coverage (mrrc) in ingroups defines the relative number of times that a SNP variant of interest had to be sequenced in ingroup and facultative ingroup respectively. Maximum absolute noise read coverage (mnrc) is the number of times that a SNP variant of interest is allowed to be sequenced in outgroup and facultative outgroup respectively. The four remaining parameters are minimum number of ingroup hits (ni), the number of genotypes in the ingroup that need to show a SNP variant and equivalent parameters for outgroup (no), facultative ingroup (nfi), and facultative outgroup (nfo). Every SNP is evaluated according to the filtering set by the authors. To identify SNP variants of interest using TME204 germplasm, we used TME204 F1-2, −7, and −8 as ingroup, and TME204 F1-1, −3, −4, −5, and −6 as outgroup and left facultative groups blank. Parameters were set to mtrc = 20, mnrc = 2 (10%), mrrc = 0.2, ni = 3, no = 5, nfi = 0 and nfo = 0. To shortcut the parameter settings and produce the results of the manuscript directly, use option -s TME204. To find SNP variants of interest for TME14, we used TME14 F1-1, −3, −5, and −6 as ingroup, TME14 F1-2, −4 as outgroup and 60444 friable embryogenic callus (FEC) Plant A, FEC Plant B, TME3 FEC A, FEC B, TME7 FEC, TME7 FEC Plant A, FEC Plant B, TME8 OES Plant A, OES Plant B, TME9 OES Plant A, OES Plant B, TME204 OES Plant, TME204 F1-1, −3, −4, −5, −6, TME419 FEC Plant A, and FEC Plant B. Parameters were set to mtrc = 20, mnrc = 2 (10%), mrrc = 0.2, ni = 4, no = 2, nfi = 0, and nfo = 9. To shortcut: -s TME14. To find the SNPs for TMS-9102324, ingroups were defined to be TMS-9102324 WT respectively. Outgroup was defined to be 60444 WT, TME14 F1-2, −4, TME204 F1-1, −3, −4, −5, and −6. No facultative ingroup was defined and the facultative outgroup consisted of 60444 FEC Plant A, FEC Plant B, TME3 FEC Plant A, FEC Plant B, TME7 FEC, FEC Plant A, FEC B, TME8 OES Plant A, OES Plant B, TME9 OES Plant A, OES Plant B, TME204 OES Plant, TME419 FEC Plant A, FEC Plant B. Parameters were set to mtrc = 20, mnrc = 2(10%), mrrc = 0.2, ni = 1, no = 8, nfi = 0, and nfo = 12. To shortcut: -s 91-02324.

### Rough genetic mapping

Genotyping by Sequencing and in silico bulk segregant analysis: Approximately 1,300 individual F_1_ progeny and the parental lines from the NASE14×TME204-LCR population generated in Kenya were characterised with genotyping-by-sequencing (GBS) at UW-Madison Biotechnology Center following their standard ApeKI restriction enzyme protocol. Reads were demultiplexed into sample fastq files using GBSX v1.3^57^ and mapped to the TME204-hap1 assembly. The GATK4 best practices pipeline^58,59^ was followed with one GBS pertinent modification (alignments were not deduplicated) to call SNPs vs the assembly. Using vcftools v0.1.14^60^, the SNPs from the parental lines (NASE14 and two TME204-LCR lines) were extracted from the quality filtered (‘QD < 2.0, QUAL < 30.0, SOR > 3.0, FS > 60.0, MQ < 40.0, MQRankSum < −12.5, ReadPosRankSum < −8.0’) VCF file and filtered to extract only those which are heterozygous in both parents (i.e. pseudo-testcross). The subset of F1s derived from these parental lines (n = 1,295) was extracted and only the pseudo-testcross positions established above were retained using bcftools^61^ ‘isec -n = 2 -w 1’ between the two VCF files. Finally, the population wide pseudo-testcross set was filtered for quality and missingness using vcftools (‘-- minDP 5, --minGQ 20, --max-missing 0.7’).

The VCF was then parsed into a tab-delimited file using GATK VariantsToTable and imported into R for further analysis. The phenotype data for each line were imported and lines were designated as resistant or susceptible as described above. A sample of 125 of the most CMD resistant and most susceptible (Resistant, both years’ disease rating = 1; Susceptible, both years’ disease rating > = 4) lines were randomly selected as the Resistant and Susceptible Bulks, respectively, to perform the in silico bulk segregant analysis using the QTLseqr package^25^. For each SNP, the mean alternative allele ratio (SNP-index) for each bulk was calculated from all the individuals in the bulk and the difference in allele ratios was compared between the two bulks (ΔSNP-index). A 5 Mb window tricube-smoothed ΔSNP-index was compared to the 95% confidence interval as in Takagi et al.^24^. SNPs with ΔSNP-index values surpassing the 95% confidence interval are significantly linked to the resistance phenotype.

### Fine-mapping

To further narrow the CMD2 locus, individual F_1_ progeny were analysed for recombination events within the defined locus (~5–13 Mb). While mapping in outcrossers using F_1_ populations is established, mapping in this population is complicated by the TME204-LCR parent in that heterozygous progeny can be either resistant or susceptible. Thus, only recombinants with a genotype-phenotype mismatch were selected as informative. For example, in a phenotypically resistant F_1_ line with a recombination that transitions from genetically heterozygous to genetically homozygous susceptible, one can exclude the homozygous susceptible region as not carrying *CMD2*. Six resistant and six susceptible recombinant individuals were identified with such recombination within the broad CMD2 locus and were used to exclude genomic regions in which at least two lines supported such exclusion.

The narrow locus defined by GBS (Chromosome12: 8,976,221-9,314,764) was used to design KASP markers (Supplementary Data 4) spanning 1.5 Mb bracketing this region. Pseudo-testcross positions were then identified by aligning WGS reads from both NASE14 and a TME204-LCR line (TME204-OES Plant, Supplementary Data 2) to the TME204-hap1 assembly and examining the reads in the two parental lines and selecting heterozygous locations which have high complexity and minimum 30% GC content in the 100 bp surrounding the SNP. Primers were then designed by IDT using their PACE/KASP marker submission form (Supplementary Data 4).

A second ~1000 individual F_1_ (NASE14×TME204-LCR) population was then screened using the highly accurate KASP-marker-based assay combined with phenotyping with a VIGS-based approach^26^. Briefly, F_1_ progeny seeds were germinated in a growth chamber at DDPSC, transferred to the greenhouse and inoculated with a virus-induced-gene-silencing version of *East African cassava mosaic virus* K201 (SPINDLY-VIGS), as described by Beyene et al. (2017)^26^. Plants were assessed over a four-week period. Plants which died were scored as CMD susceptible while those that recovered from initial symptoms and re-established healthy growth were scored as CMD resistant. Additional recombinants within the second population were sought in a similar manner as above.

The standard KASP protocol was used to genotype every individual in the fine-mapping population on a BioRad CFX384 using the Allelic Discrimination tab in the CFX software package. The full population was screen with markers M1, M2 and M6, M8 and recombinants between these markers were further screened using the markers within that interval (M3, M5, M7). The original marker numbering scheme represents their order based on the AM560-2 ref 6.1 assembly; however, the positions have been updated to reflect the more accurate positions in the TME204-hap1 assembly^23^. The list of recombinants was narrowed to only those with phenotype-genotype mismatch and a minimal recombination site was identified as linked to the phenotype. To confirm these results, 5–7 replicates of each line were regenerated from tissue culture and re-phenotyped using the above methods. The genotypes of the regenerated lines were also confirmed with all KASP markers, and the recombinant lines and controls were sequenced using Illumina as above and nucleotide level comparison was performed by alignment to TME7^22^ and TME204^23^ assemblies and manual inspection using CLC Genomics and IGV^54^.

### RNAseq and differential expression analysis

Two RNAseq experiments were performed comparing resistant to susceptible samples. In experiment 1, leaf samples from TME204-WT and TME204-LCR plants (FEC- derived) were compared. Three cloned plants of TME204-WT and three cloned plants of each of three FEC-derived plants were sampled such that a total of 3 resistant and 9 susceptible plants were used in the experiment. One replicate of one of the FEC lines failed so the total samples sequenced was 11 not 12. In RNAseq experiment 2, leaves were collected from a selection of resistant and susceptible F_1_ lines (derived from a TME204-WT self-cross). To multiply the plant material, cuttings from each plant were first collected in advance of phenotyping. Plant lines were then assessed for their resistance phenotype using the above VIGS approach. For the RNA sample collection, two uninoculated clonal plants from each of three resistant and susceptible F_1_s were used.

For both experiments, the youngest fully expanded leaves were harvested from plants 4–5 weeks after transplanting to soil. Samples were collected and frozen in liquid nitrogen until RNA extraction with the Sigma Spectrum Plant Total RNA Kit. RNAseq was performed after poly-A selection using Illumina Hi-Seq 2000 (2 × 101 Paired end reads) and Hi-Seq 3000 (2 × 150), for experiments 1 and 2 respectively. Sequencing was performed at Washington University in St Louis Genome Technology Access Center.

For differential expression analysis, first a transcriptome fasta of the spliced exons was made from the TME204-hap1 gff file using ‘gffread -w’ from the cufflinks package^62^. This transcriptome was then concatenated to the whole genome to prepare an alignment decoy file and index using the commands here https://combine-lab.github.io/alevin-tutorial/2019/selective-alignment/. Trimmed RNAseq reads were then pseudo-aligned to the TME204-hap1 transcriptome using Salmon v1.5.2 default settings^63^. Read count data were imported into R using the tximport package^64^. Samples were then defined as resistant or susceptible and differential expression on the integer count values was performed using DESeq2^65^. Genes with a sum of less than 50 reads across all samples were excluded from analysis. Differential expression was performed using “apeglm” as the Log Fold Change Shrinkage method^66^. Genes were defined as being significantly differentially expressed if they had an adjusted *p*-value^67^ of less than 0.05. Normalised counts were plotted using ggplot and tidyverse^68^ functions in R.

### Monte Carlo sampling

After performing the SNP analysis, the number (n) of SNPs leading to an amino acid change was counted for the given scenario. Next, we randomly chose n bp positions throughout all 18 chromosomes of the TME204 genome and marked them as being hypothetical SNPs. If at least one SNP was present within the defined locus (between marker M3 and M7), we identified this iteration of the experiment to have yielded success. Otherwise, the round was counted as being unsuccessful. We repeated this experiment 100’000 times and the ratio of successes represents a rough estimate of the likelihood that an amino acid changing SNP is found by chance within the locus.

### Virus-induced gene silencing (VIGS) targeting of *MePOLD1*

VIGS vector construction and plant inoculation: The VIGS-based screening method developed by Lentz et al.^34^ was used to study the effects of the gene of interest on CMD resistance. A 400 bp coding sequence of *MePOLD1* (position 438-837, corresponding to 8905307-8905965 of chr12 in AM560 v8, 9076083-9076741 of chr12 in TME204-hap1) was synthesised (Twist Biosciences, California, USA) and inserted in the multiple cloning site of the ACMV-based VIGS vector using *Kpn*I and *Spe*I. The 400 bp coding sequence is conserved in *MePOLD1* of 60444, TME3, TME204 and AM560. n-mers (18–24 nt) with zero mismatches were checked against the cassava AM560 v6.1 genome sequence with SGN VIGS from Sol Genomics (https://vigs.solgenomics.net/)^38^ to validate that the sequence selected to target *MePOLD1* has no off-targets in the cassava genome. The number of 60444 plants inoculated were *n* = 15 for ACMV, *n* = 40 for *MePOLD1*-VIGS, *n* = 30 for *GUS*-VIGS, and *n* = 15 for mock treatments. Leaf symptom scoring was based on the 0-5 scale as described by Fauquet and Fargette (1990)^56^.

ACMV titre quantification: Total DNA was extracted from the youngest 1-2 leaves. Leaves were harvested at first signs of CMD symptoms and snap frozen in liquid nitrogen with the DNeasy Plant Mini Kit (QIAGEN, Germany). Quality was assessed by Nanodrop (Thermo Scientific, Wilmington, USA) and quantified with Qubit dsDNA BR Assay Kit (Thermo Fisher Scientific Inc, Massachusetts, USA). ACMV titre was quantified with qPCR using the LightCycler 480 System (Roche) with 15 ng of total DNA, 1 μM of primers, and Fast SYBR Green Master Mix (Applied Biosystems, Massachusetts, USA) in a final volume of 10 μL. ACMV DNA-A specific primers and the endogenous cassava *PP2A* gene (*Manes.09G039900*) were used as an internal control (Supplementary Table 2) with at least three technical replicates were included per sample. A two-tailed Mann-Whitney U test was used to analyse the statistical significance. Primers are listed in Supplementary Table 2.

Gene expression analysis: Total RNA was extracted from the top 1-2 leaves. Leaves were harvested at first signs of CMD symptoms and snap frozen in liquid nitrogen with the Spectrum Plant Total RNA Kit (Sigma-Aldrich, Merck Life Science, Germany) according to Protocol A. An On-column DNAase I Digestion (Sigma-Aldrich, Merck Life Science, Germany) was performed as manufacturer’s instructions to remove residual genomic DNA. RNA quality was assessed with the Nanodrop system (Thermo Scientific, Wilmington, USA) and quantified with Qubit RNA BR Assay Kit (Thermo Fisher Scientific Inc, Massachusetts, USA). The samples were converted to cDNA using the RevertAid First Strand cDNA Synthesis Kit (Thermo Scientific, Wilmington, USA) according to the manufacturer’s instructions. *MePOLD1* (*Manes.12G077400*) relative expression was quantified with RT-qPCR in triplicates using the LightCycler 480 System (Roche) with 15 ng of cDNA, 1 μM of primers, and Fast SYBR Green Master Mix (Applied Biosystems, Massachusetts, USA) in a final volume of 10 μL. The comparative CT (threshold cycle) method^69^ was used to calculate relative transcript levels with *Tubulin 1 β chain* (*MeTUB1*, *Manes.08G061700*) as the reference gene. Primers are listed in Supplementary Table 2.

### Identification of additional MePOLD1 variants

A publicly available dataset was accessed containing sequencing data of 241 diverse accessions that identified over 28 million segregating variants^37^. All positions within the *MePOLD1* gene (AM560-2 v6.1 coordinates) were extracted from the Chromosome12 VCF file available through the cassavabase.org FTP server (c12.DepthFilt_phasedSNPs.vcf), and the effects of the variants on the protein coding sequence determined using snpEff^70^. Additional analysis was done with Sanger sequencing (Supplementary Data 6). Names listed in Fig. 4c are as listed in Ramu et al.^37^ We note that according to this publication, TMS972205 contains a different SNP than the one identified here and is referred to as TMS-972205.

### POLD1 Protein sequence analyses

The 3D structure of the yeast POLD catalytic subunit and template DNA (PDB ID: 3IAY) was visualised in ChimeraX^71^. The N-terminal domain, exonuclease domain, and finger, palm, and thumb motifs from Swan et al., 2009^72^ were colour-coded and the residues corresponding to the nonsynonymous mutations identified across the cassava varieties are highlighted.

### Analysis of *MePOLD1* in 5001-NASE 14-#41

The full-length cDNA of *MePOLD1* was amplified from cassava plant line 5001-NASE 14-#41^43^. Primers were designed to be specific for the haplotype carrying the resistance *MePOLD1* allele and PCR performed. The PCR product was cloned into the binary vector pCAMBIA1305.1 using the In-Fusion^®^ HD Cloning Kit (Takara Bio USA, Inc.) and the resulting clones sequenced by Sanger sequencing. Primers are listed in Supplementary Table 2.

### Reporting summary

Further information on research design is available in the Nature Research Reporting Summary linked to this article.

## Supplementary information

Supplementary Information

Description of Additional Supplementary Files

Supplementary data 1

Supplementary data 2

Supplementary data 3

Supplementary data 4

Supplementary data 5

Supplementary data 6

Reporting Summary

## Data Availability

Source data are provided with this paper as Supplementary Datasets. Raw bisulfite sequence data is available through NCBI GEO GSE192748. Whole Genome Sequencing and RNAseq raw read data can be accessed at NCBI sequence read archive PRJNA787456. Source data are provided with this paper.

## Source data

Source Data

## Code availability

Scripts for WGS-GVA are deposited in github [https://github.com/pascalschlaepferprivate/filter_vcf]. Scripts for mapping analyses are here [https://github.com/bmansfeld/CMD2_project]. Other scripts available in Source Data.
