# Supplementary material for: Mutations in DNA polymerase δ subunit 1 co-segregate with CMD2-type resistance to Cassava Mosaic Geminiviruses
Source: Nat Commun. 2022 Jul 7;13:3933. doi: 10.1038/s41467-022-31414-0 (PMC9262879; doi:10.1038/s41467-022-31414-0)
Supplement: Supplementary file 1 — Supplementary Information [file 41467_2022_31414_MOESM1_ESM.pdf]

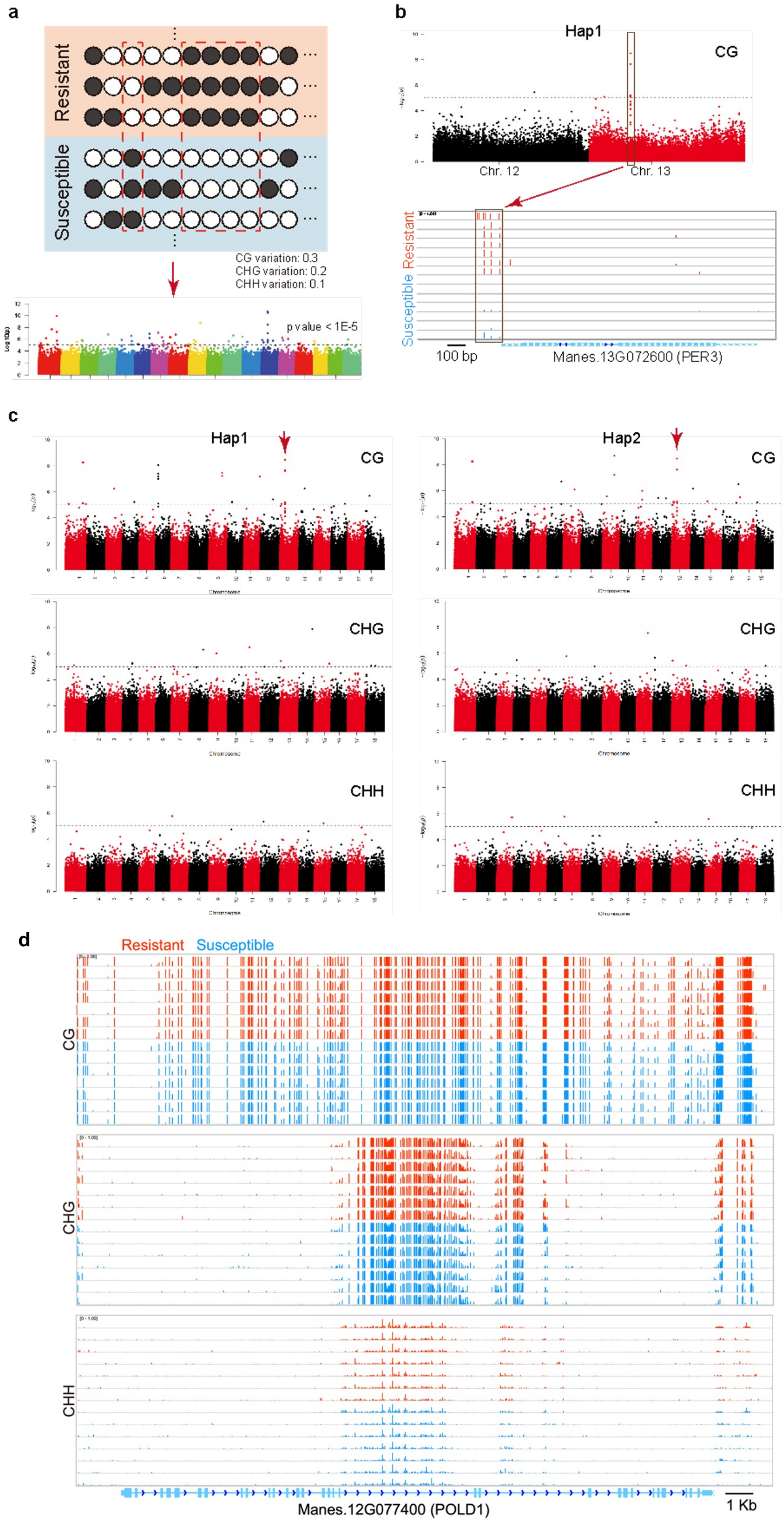

Supplementary Fig. 1. Full genome bisulfite sequencing of cassava varieties before and after tissue culture induced *de novo* morphogenesis. A) Analysis strategy cartoon. B) A single peak on chromosome 13 was identified as differentially methylated between resistant and susceptible lines. C) differential methylation across two haplotypes (Qi, W. et al 2021) for CG, CHG and CHH methylation. One-way ANOVA test has been used to identify significantly ($p < 0.05$) differential methylated cytosines (CG > 0.3, CHG > 0.2, and CHH > 0.1) between resistant and susceptible samples. D) *Me POLD1* does not display differential methylation between resistant and susceptible cassava lines.

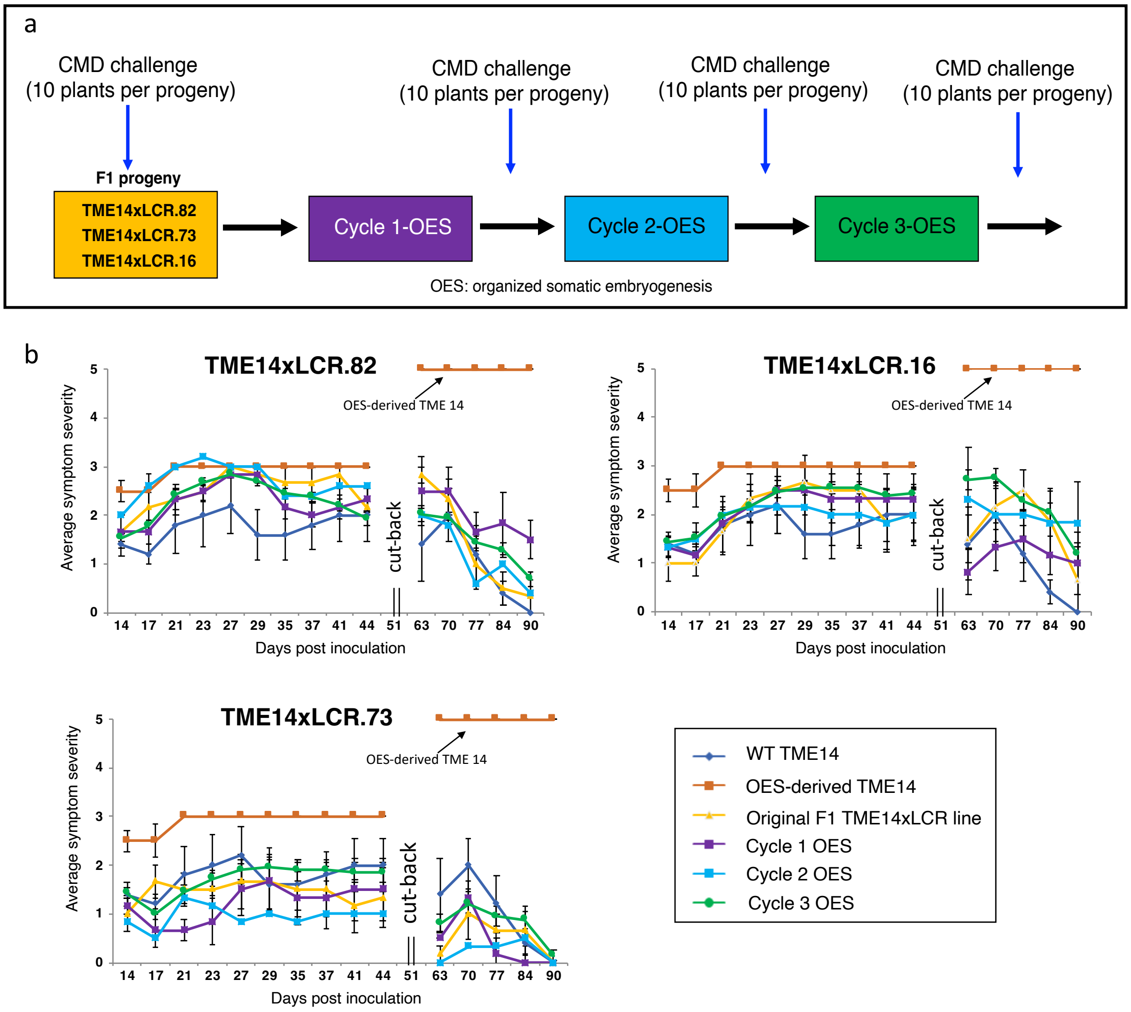

Supplementary Fig. 2. CMD2-mediated resistance remains stable in cassava progeny lines generated through sexual crosses. TME204-LCR lines were crossed with TME14 which carries functional CMD2-type resistance. a. Three progeny lines were passed through successive cycles of somatic embryogenesis. Plants were regenerated after each cycle and inoculated with the virulent EACMV isolate K201. b. CMD leaf symptoms were assessed visually on a 0-5 scale over 44 days, after which plants were ratooned and new growth scored until 90 days after inoculation. TME14 wildtype plants (blue, identical data shown in all three panels) displayed the recovery phenotype typical for this landrace, while OES-derived TME14 (LCR) lines passed through embryogenesis (orange, identical data shown in all three panels) became highly CMD susceptible, displaying the highest disease severity (5) and no recovery (arrowed). Plants regenerated from TME14xLCR F1 progenies remained resistant to CMD at a level not significantly different from the original F1 progeny line from which they were derived. Ten replicate plants were challenged for each line. Error bars are standard deviation from the mean. OES: organized embryogenic structures

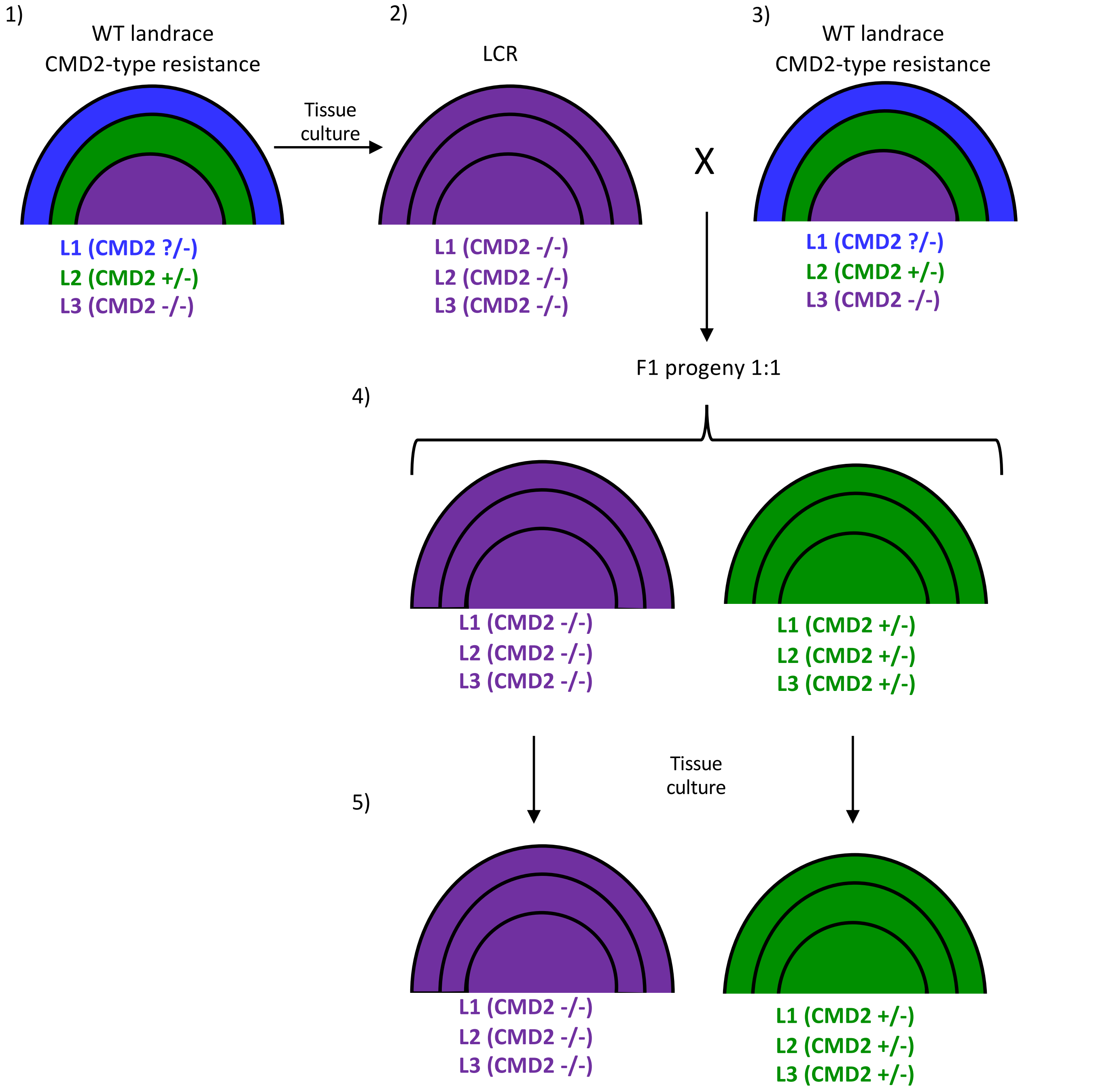

Supplementary Fig. 3. In the model cartooned below, L2 contains the resistant allele (green) while L3 does not (purple) (1). L3 gives rise to embryogenetic tissue (Loss of CMD2 Resistance - LCR) through *de novo* morphogenesis that is non chimeric and lacks the resistance allele (2). A similar scenario is possible if the L1 is -/- for CMD2 and gives rise to embryogenic tissue. When an LCR plant is crossed with a chimeric wildtype (WT) CMD2-type cassava variety, F1 progeny match the genotype of the parental L2 layer and therefore segregate resistance 1:1 and are not chimeric (4). Consequently, when resistant and susceptible F1 progeny are passaged through tissue culture induced *de novo* morphogenesis, the maintain their resistant or susceptible phenotypes, respectively.

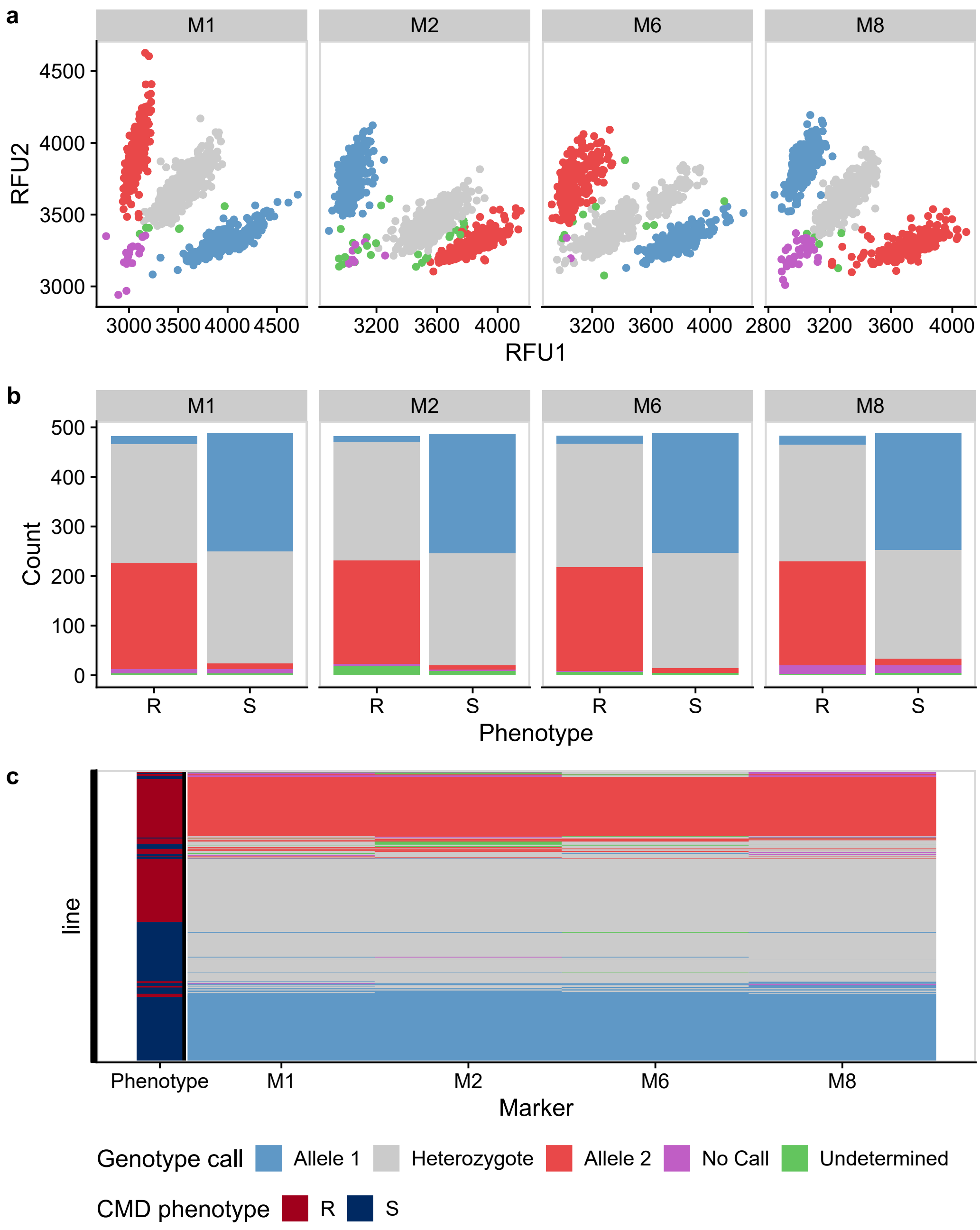

Supplementary Fig. 4. Genotyping using Kompetitive Allele Specific PCR (KASP) markers. a) The entire ~1000 F1 population was screened using 4 KASP markers, M1, M2, M6, and M8. Dot plot of the raw relative fluorescence units (RFU) for the two allele specific primers. Each point is an individual F1 progeny and the colours represent the allele call made for each marker. b) The distribution of genotype calls for each of the two phenotypic states (R - Resistant, S - Susceptible). c) A view of the all the calls of the entire ~1000 individual population. The markers on the x-axis are ordered by their genomic position allowing to visualize recombinants between the markers. The resistance phenotype is indicated on the left bar.

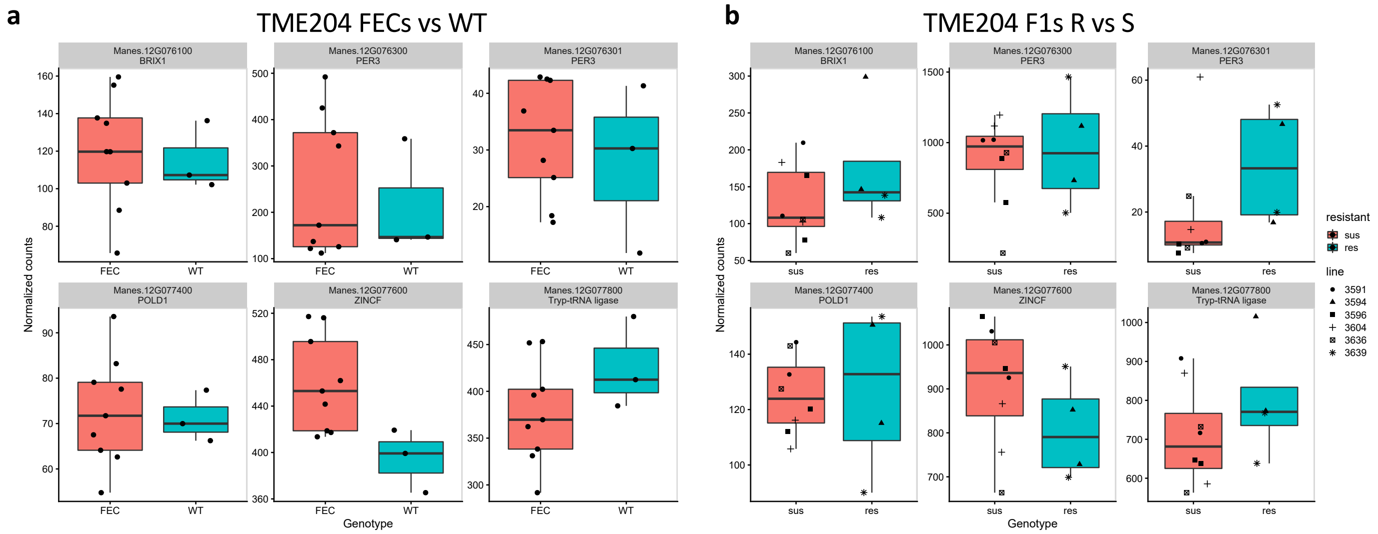

Supplementary Fig. 5. Gene expression analysis of genes within the fine-mapped CMD2 locus. (A) Resistant TME204 wildtype plants were compared to susceptible TME204-LCR plants regenerated through embryogenesis during production of friable embryonic callus (FEC). (B) Resistant and susceptible F1 plants derived from a TME204-WT self-cross were compared. Of the 8 genes defined within the 190Kb locus, only 6 are detected as expressed in our datasets. BRIX1 - RIBOSOME BIOGENESIS PROTEIN BRIX; PER3 – PEROXIDASE 3-RELATED; POLD1 - DNA POLYMERASE DELTA; ZINCF - ZINC FINGER, CCCH-type. The data are presented as standard boxplots (the box encompasses Q1–Q3, the median is shown as a central horizontal line within the box, and the whiskers cover the data within ±1.5 IQR).

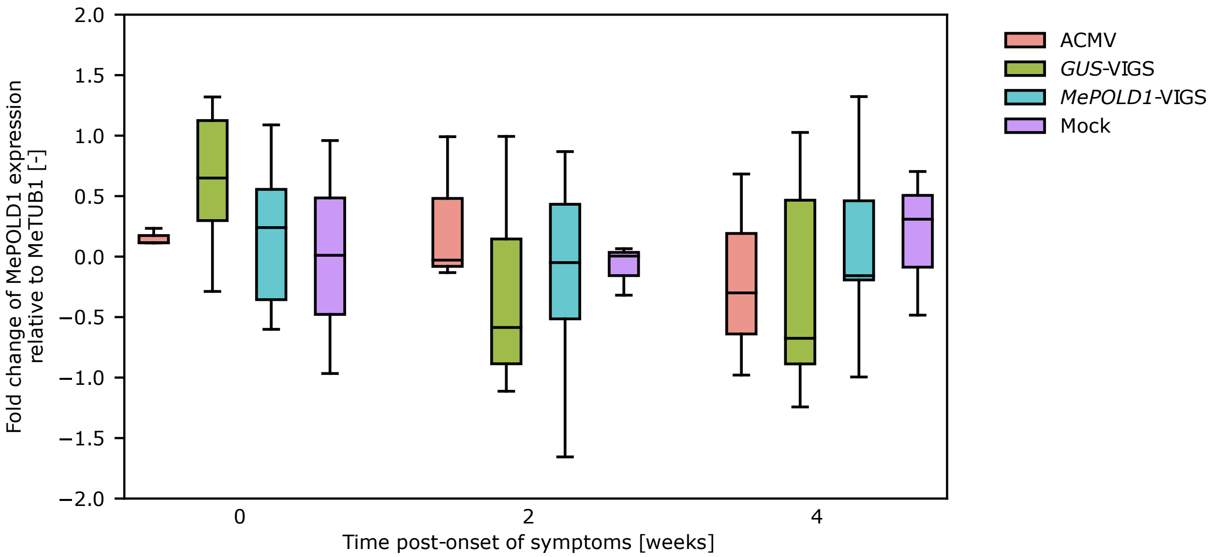

Supplementary Fig. 6. *MePOLD1* expression relative to expression of the gene encoding *Tubulin 1 β chain* (*MeTUB1*, Manes.08G061700) after ACMV-VIGS inoculation (non-modified ACMV, *GUS*-VIGS, *MePOLD1*-VIGS and mock) of CMD- susceptible cassava 60444. Week 0 is the first onset of symptoms of individual plants and week 2 is two weeks after that and so on. Number of biological replicates at Week 0, 2 and 4 respectively: ACMV (n = 3, 3, 3), *GUS*-VIGS (n = 10, 10, 8), *MePOLD1*-VIGS (n = 9, 10, 9), and mock (n = 3, 3, 3). The data were presented as standard boxplots (the box encompasses Q1–Q3, the median is shown as a central horizontal line within the box, and the whiskers cover the data within ±1.5 IQR).

MePOLD1

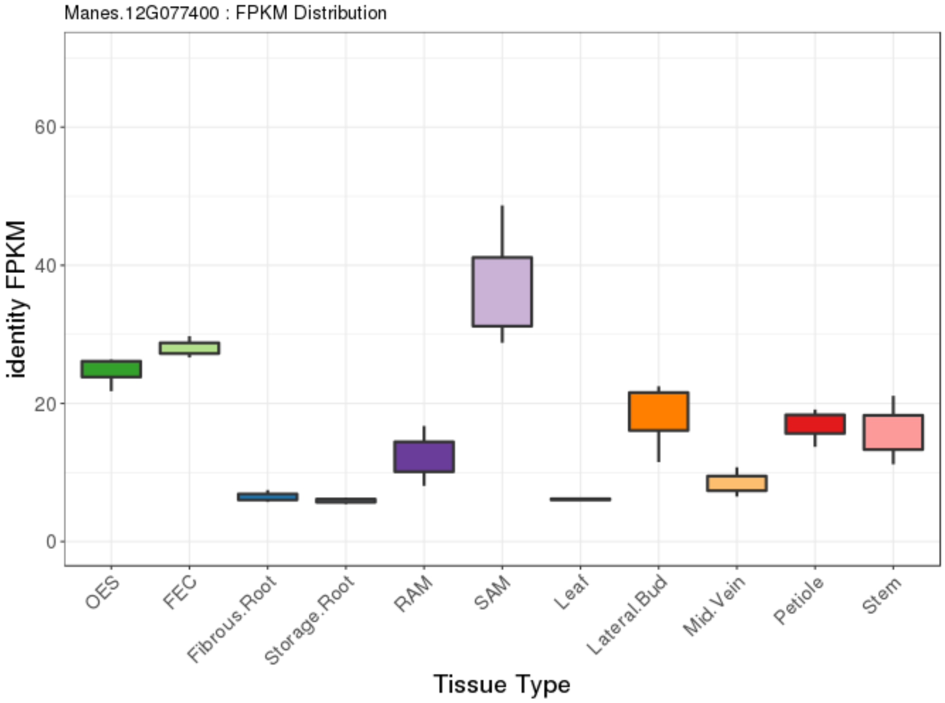

MeTUB1

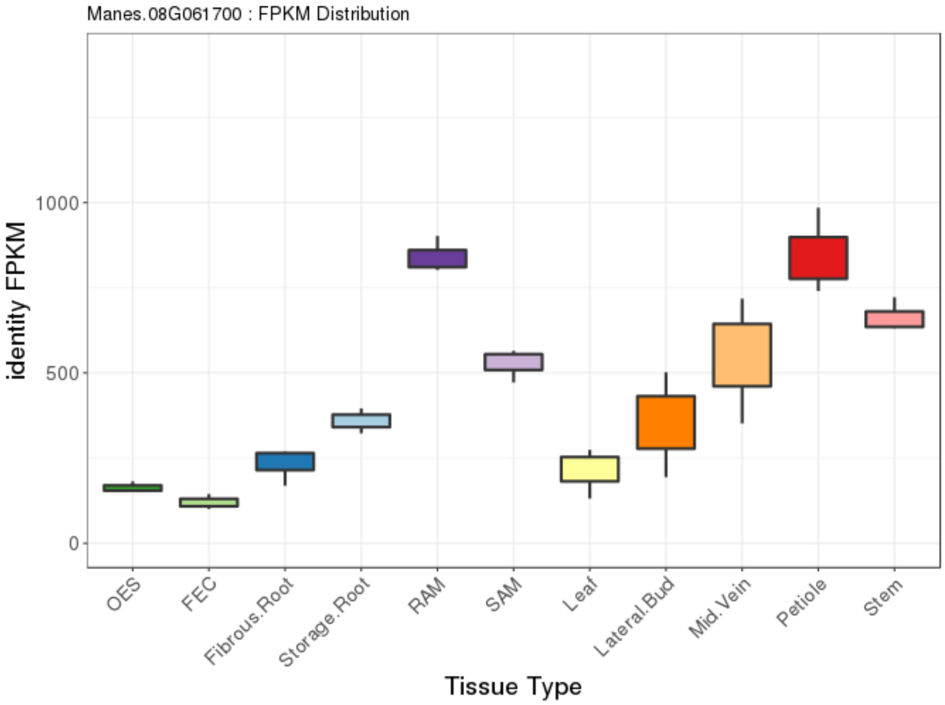

Supplementary Fig. 7. Gene expression across multiple tissue types. Data obtained from the Cassava Expression Atlas (Wilson et al. 2017). The data are presented as standard boxplots (the box encompasses Q1–Q3 and the whiskers cover the data within ±1.5 IQR).

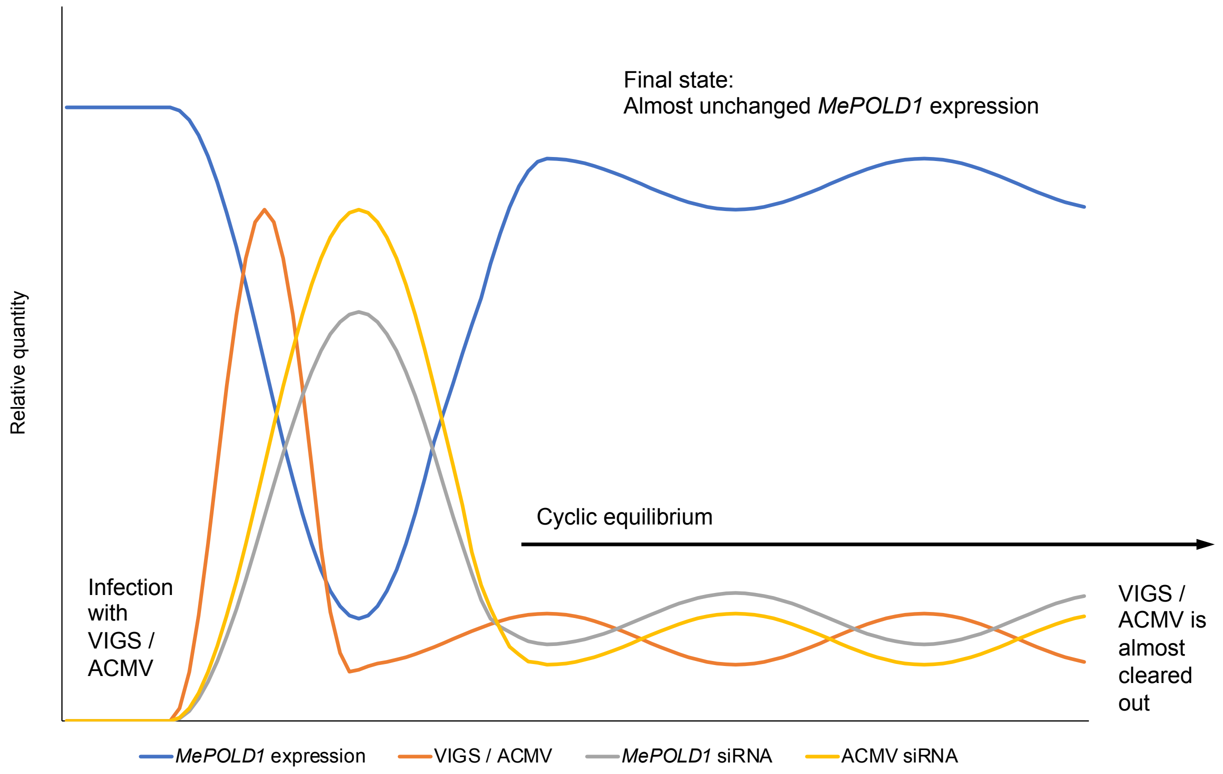

Supplementary Fig. 8. Hypothesis to explain the observed *MePOLD1* expression pattern after ACMV-VIGS inoculation and reduction of ACMV load in CMD-susceptible 60444**.** The blue line represents the hypothetical *MePOLD1* expression and it starts off under normal conditions. Once the plant has been inoculated with the *MePOLD1* VIGS construct which is a modified ACMV clone (represented by the orange line – VIGS/ACMV), the rise of VIGS/ACMV leads to the reduction (increase in *MePOLD1* siRNA represented by the grey line as well as siRNA of ACMV represented by the yellow line) of *MePOLD1* expression. Since ACMV needs MePOLD1 to replicate, as *MePOLD1* expression drops and ACMV siRNA increases, the quantity of VIGS/ACMV will also decrease. The reduction in VIGS/ACMV then leads to lower production of siRNA against *MePOLD1* thereby allowing the expression of *MePOLD1* to return to approximately normal quantities. Since *MePOLD1* level returns close to normal, VIGS/ACMV will also begin increase in quantity. However, since there are residual amounts of ACMV siRNA, VIGS/ACMV will never be able to establish itself thus leading to a cyclic equilibrium where VIGS/ACMV is maintained at a low quantity and *MePOLD1* expression remains almost unchanged.

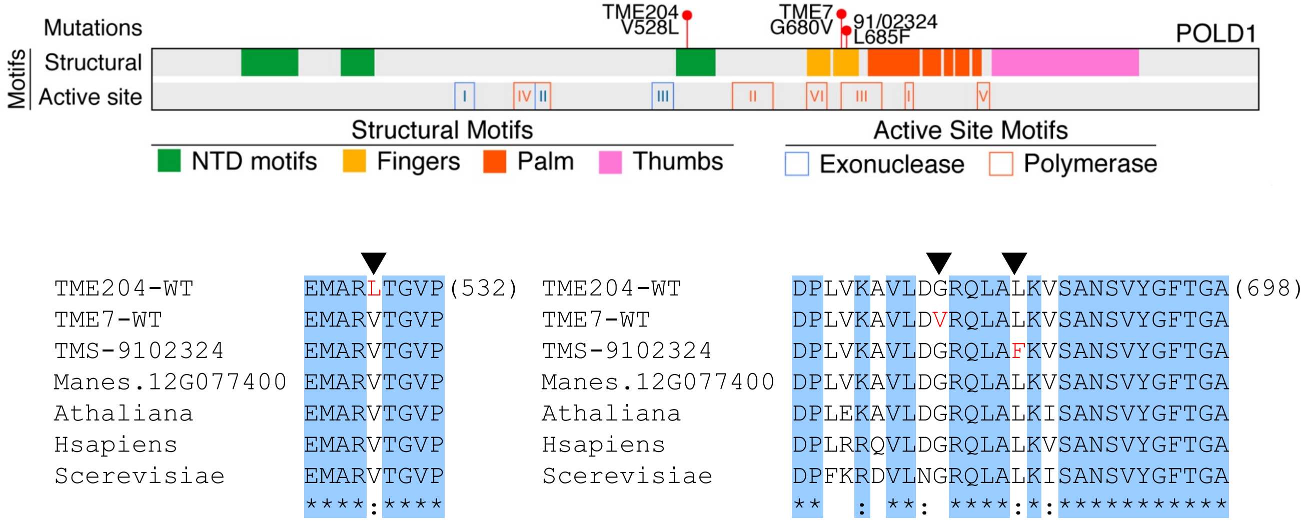

Supplementary Fig. 9. (Top) Schematic diagram of the POLD1 protein from cassava. Red lollipop flags indicate locations of resistance alleles. Active site motifs in the exonuclease and polymerase domains are indicated by the blue and orange outlined boxes, respectively. (Bottom) Protein alignment of POLD1 sequences. Sequences from three varieties containing a non-synonymous SNP are included. Affected amino acid is noted by an arrow head and the mutated residue in red; position of the last amino acid in alignment is indicated in paratheses. Manes.12G77400: *Manihot esculenta* AM560-2 v6.1; Athaliana: *Arabidopsis thaliana*; Hsapiens: *Homo sapiens*; Scerevisiae: *Saccharomyces cerevisiae*.

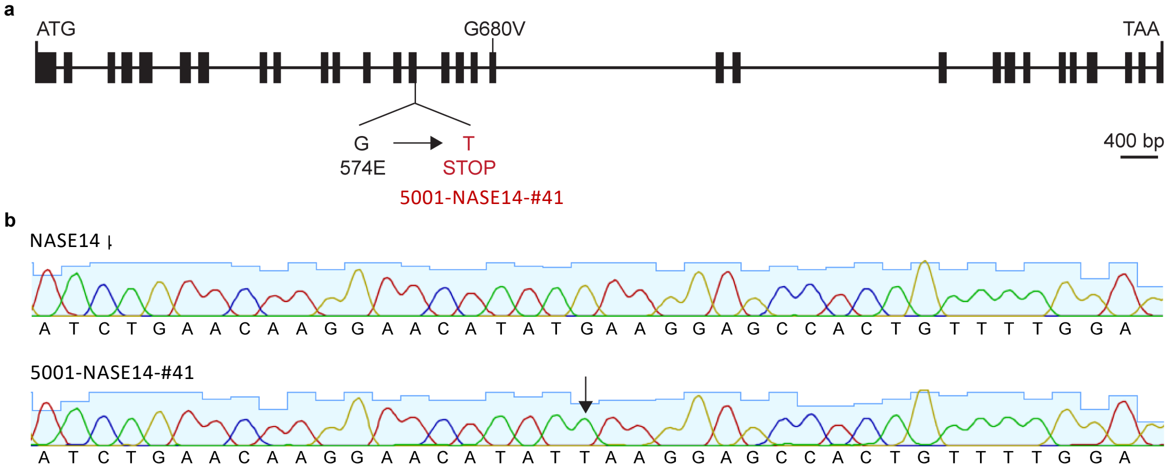

Supplementary Fig. 10. Premature stop codon within the resistant haplotype of *MePOLD1* in susceptible line 5001-NASE14-#41. 5001-NASE14-#41 is a transgenic line from resistant NASE14. **a**, Schematic diagrams show the gene structure of the resistant haplotype of *MePOLD1.* The exons are indicated as solid boxes, and the introns are indicated as lines. The mutation site in the resistant haplotype of *MePOLD1* in line 5001-NASE14-#41 is highlighted in red. **b**, Sanger sequencing analysis identified a mutation in the resistant haplotype of *MePOLD1* in line 5001-NASE14-#41. The full-length cDNA sequence was amplified by a pair of primers which specifically worked for resistant haplotype of *MePOLD1* in resistant NASE14 and its derived lines.
