# Supplementary material for: Mutations in DNA polymerase δ subunit 1 co-segregate with CMD2-type resistance to Cassava Mosaic Geminiviruses
Source: Nat Commun. 2022 Jul 7;13:3933. doi: 10.1038/s41467-022-31414-0 (PMC9262879; doi:10.1038/s41467-022-31414-0)
Supplement: Supplementary file 2 — Description of Additional Supplementary Files [file 41467_2022_31414_MOESM2_ESM.pdf]

Description of Additional Supplementary Files

File Name: Supplementary Data 1
Description: Field level disease scores for mapping population individuals.

File Name: Supplementary Data 2
Description: List of lines used in this study.

File Name: Supplementary Data 3
Description: SNP calls from WGS.

File Name: Supplementary Data 4
Description: KASP marker details.

File Name: Supplementary Data 5
Description: Greenhouse level disease scores for mapping population individuals.

File Name: Supplementary Data 6
Description: Summary Table for identified SNPs in MePOLD1.
