# Supplementary figures and images for: Mutations in DNA polymerase δ subunit 1 co-segregate with CMD2-type resistance to Cassava Mosaic Geminiviruses
Source: Nat Commun. 2022 Jul 7;13:3933. doi: 10.1038/s41467-022-31414-0 (PMC9262879; doi:10.1038/s41467-022-31414-0)

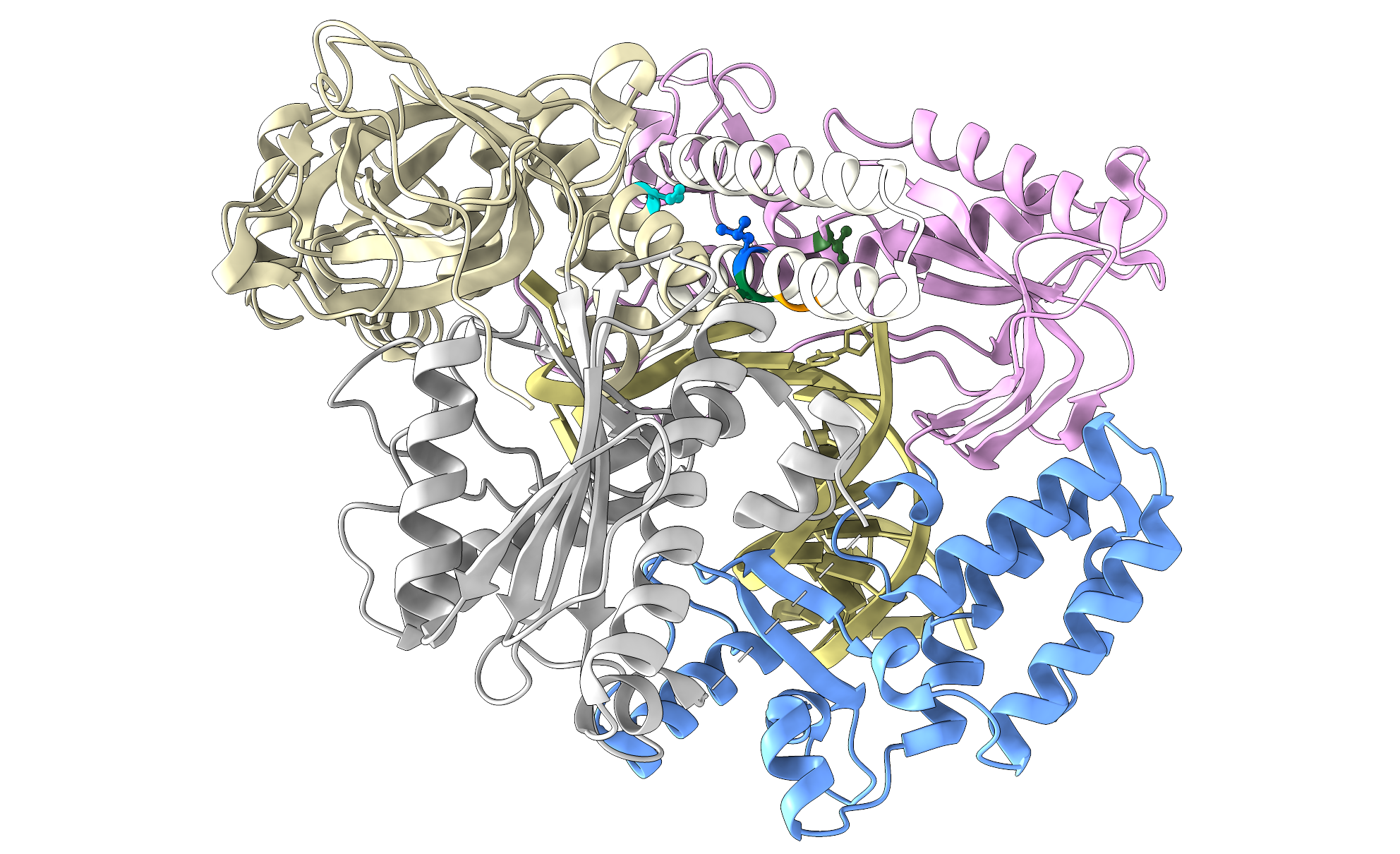

Supplement: Supplementary file 10 — Source Data [file 41467_2022_31414_MOESM10_ESM.zip › figure_data/Figure4b_c_d_e_data/Figure4b_c_d_e/Fig4d-3IAY-color_coded_labeled.figure.postReview-angle1.png]

Variety

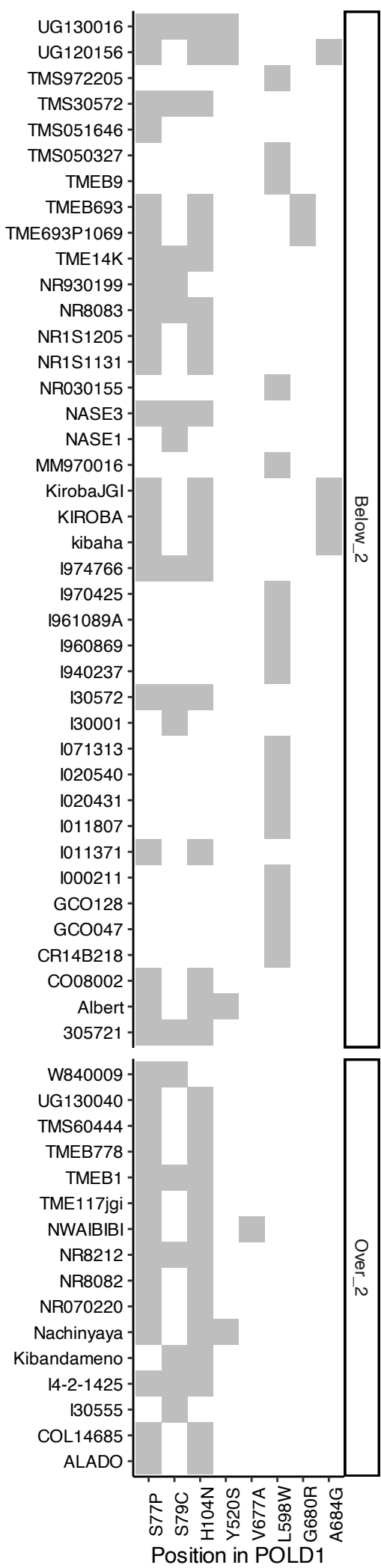

Supplement: Supplementary file 10 — Source Data [file 41467_2022_31414_MOESM10_ESM.zip › figure_data/Figure4b_c_d_e_data/Figure4b_c_d_e/Fig4c-Other_SNPs.CMD_data.pdf]

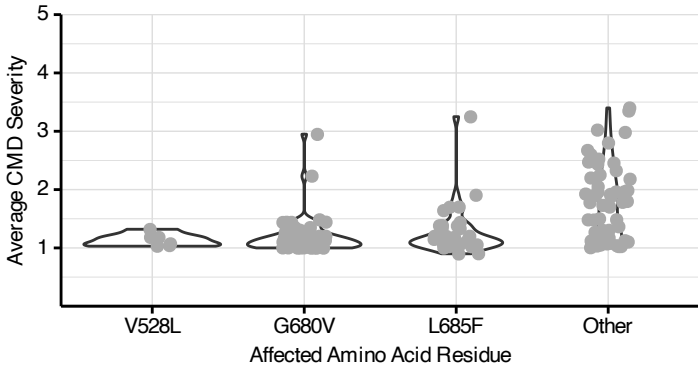

Supplement: Supplementary file 10 — Source Data [file 41467_2022_31414_MOESM10_ESM.zip › figure_data/Figure4b_c_d_e_data/Figure4b_c_d_e/Fig4b-summary.hapmap.3SNPs.other.CMDdata.violinPlot.pdf]
